# The assessment and management of acute trauma pain in a Cape Town, South Africa Emergency Centre: A retrospective chart review

**DOI:** 10.1016/j.afjem.2026.100949

**Published:** 2026-02-07

**Authors:** Ngcebo Ndebele, Laverne Phillips, Peter Hodkinson

**Affiliations:** a Division of Emergency Medicine, Department of Family, Community and Emergency Care, University of Cape Town, Cape Town, South Africa; b Mitchells Plain District Hospital, Department of Health, South Africa

**Keywords:** Pain, Acute trauma, Pain assesment and management, Anaglesia, Emergency

## Abstract

**Background:**

Trauma accounts for over 60,000 deaths annually in South Africa and is also responsible for a high proportion of emergency centre (EC) visits. Up to 91% of trauma patients in the EC experience acute pain, underscoring a critical public health concern, particularly in low and middle-income countries (LMICS), where research indicates poor pain management and a paucity of data. This study aimed to describe trauma pain assessment and management practices in a busy Cape Town EC.

**Methods:**

This single-centre retrospective chart review in a high trauma burden EC collected a convenience sample over two weeks in 2024. Data were extracted from the medical records of adult trauma patients presenting to the EC.

**Results:**

A total of 234 patients were included, predominantly male (73.1%), with a median age of 33 (IQR 26–41). Only 32.9% (77) of the patients had their pain assessed and documented, largely only in the triage process, and no patient had their pain reassessed. Furthermore, only 42.3% (99) of patients received analgesia, including opioids administered to 48.5% (48), non-steroidal anti-inflammatories to 45.5% (45), paracetamol to 69.7% (69), and ketamine to 17.2% (17). The time from arrival to the administration of the first analgesia in the EC was documented in 83 patients, with a median time to analgesia of 375 min (IQR 152–611).

**Conclusion:**

This study demonstrates findings consistent with the global crisis of inadequate pain management. It reveals poor pain assessment and management, with prolonged waiting times for analgesia despite various indicators of potential pain severity, highlighting a crucial need for changes in pain management strategies. Training, awareness, and protocols to improve pain management are essential, along with considering nurse-led analgesia at triage.

## African relevance

• Pain is neglected in many emergency settings, probably more so in resource-poor settings.
• Access to pain management is a fundamental human right and should be prioritised even in the emergency setting.
• Pain was poorly assessed and managed in a relatively well-resourced EC with multiple available analgesics.
• We should consider alternative pain management strategies in busy ECs, such as allowing nurses to administer the first dose of analgesia during triage.

## Introduction

Injuries account for tens of millions of visits to emergency centres (EC) and other acute care settings, particularly in Low and Middle-Income Countries (LMICs), where emergency resources are often stretched and still developing in many areas [1]. Trauma remains a significant cause of morbidity and mortality, with South Africa alone witnessing over 60,000 trauma-related deaths annually [2]. Up to 70% of trauma patients in the prehospital setting and 91% in the EC setting experience pain, making it a significant public health concern [3,4]. Pain (recognised as the fifth vital sign) must be regarded as a subjective experience influenced by physiological, psychosocial, and cultural factors, and is most reliably assessed through self-reporting [5,6].

The management of acute trauma pain is known to be poorly taught and implemented in South Africa and beyond [[7], [8], [9]]. Poorly managed pain is associated with adverse physiological and psychological effects, including impaired breathing, increased metabolic demands, a weakened immune system, poor wound healing, post-traumatic stress disorder (PTSD), and developing chronic pain [5,10]. Management of acute trauma pain is a critical area of focus in Emergency Medicine (EM), especially in LMICs and needs to be prioritized in a time pressured environment.

EM is a relatively new speciality in South Africa and faces many challenges, including a high burden of trauma, which accounts for approximately one-third of emergency admissions, resource limitations, and overcrowding [11,12]. These challenges often affect the quality of patient care [13]. Pain management requires a formal assessment using validated tools and repeated evaluations to guide management [14,15].

International studies have shown that pain management in ECs is often poor due to pain being treated as a lower priority, knowledge gaps among clinicians and patients, and poor attitudes towards pain management [16,17]. Locally, several studies in a prehospital and inpatient setting have demonstrated suboptimal pain management [7,18]. Given the limited data on pain assessment and management practices in LMICs, particularly in the ECs, we aimed to describe acute trauma pain assessment and management in a Cape Town EC.

## Methods

This study was an observational, descriptive, retrospective chart review, and the data were collected from existing electronic patient records.

### Study setting

The study was conducted in the EC of Mitchells Plain District Hospital (MPH), a 230-bed district hospital located in Mitchells Plain, a suburb 32 km from Cape Town city centre, Western Cape, South Africa. MPH is estimated to serve approximately 650,000 low-to-middle-income patients, with a high trauma burden, unemployment, poverty, crime, drug abuse, and gangsterism making it one of the busiest hospitals in the Cape Town metropolitan area [19,20]. The MPH EC treats about 55,000 patients annually (of which some 10–18% are trauma-related) in a 25-bed EC that includes four resuscitation beds and is staffed by four emergency physicians, rotating EM registrars and interns, community service medical officers(COSMO), and medical officers (MOs), with 1–2 specialists, 1 EM registrar, 1 MO, 2 CSMOs, 2 intern doctors on the floor at any one time [[19], [20], [21]]. The nursing team comprises Registered nurses (RNs), specialist trauma registered nurses (RNs), enrolled nurses (ENs), enrolled nursing auxiliaries (ENAs), and caregivers, with 4 RNs,5 ENs, and 1 ENA nurse on the floor at any given time. One professional nurse and one experienced enrolled nurse attend to the triage area. Approximately 4500 patients per month visit Mitchell Plain EC, with 55% classified as high acuity (orange and red triage category).

### Study participants & sampling

All adult trauma patients presenting to MPH EC during the two-week study period from 19 February 2024 to 3 March 2024 were included. This period was chosen for convenience to include two weekends, one of which is a month-end period when trauma is known to spike [22]. The proportion of patients assessed for pain and managed appropriately in this setting is unknown; therefore, this study formed a pilot investigation, and a sample size calculation was not indicated. We estimated that a sample of 150 trauma patients over two weeks would be enough to reflect practice on most days in MPH, as a 2019 study showed that MPH attends to 332 trauma patients per month [21]. All adult patients (age 13 years or above as per local practice) who presented with acute trauma (injury not older than 1 week before the visit to EC) for their first visit related to that injury were included [23]. Exclusion criteria included repeat visits, folders with significant missing data, i.e., missing EC doctor’s consultation notes or triage forms, and patients who left without being seen.

### Data collection and management

Data were extracted from two official electronic databases that routinely track patients and their management: the Hospital and Emergency Centre Tracking and Information System (HECTIS) and Enterprise Content Management (ECM). Trauma patients who presented during the study period were identified, and their records were extracted electronically. Patient acuity is assessed using the South African Triage Scale (SATS), which incorporates verbal rating scale (VRS) pain assessment as a discriminator [24,25]. The data points collected were age, sex, SATS triage category (green- non-urgent, yellow- urgent, orange- very urgent, red- emergency), time of arrival, time of triage, pain assessment, mechanism of injuries, International Statistical Classification of Diseases and Related Health Problems,10th Revision (ICD-10) diagnosis, dispositions, mode of referral and transport, prescribed analgesia, and time of administration.

Of note, VRS pain assessment influences the triage score, as moderate pain results in a yellow triage score, while severe pain results in an orange score, meaning that a patient should be seen by a doctor within 1 hour, and 10 min, respectively [24,25]. The prescribed treatment to take out (TTO) and analgesics given after the patient left the EC for admission were not included because the study aimed to assess analgesics administered while the patient was in the EC. The study also did not include subcutaneous infiltration of local anaesthesia during wound suturing as analgesia; however, regional blocks were included.

A research assistant (medical intern) was trained by the first author on data collection and extracted, entered, and stored the data in a password-protected Microsoft spreadsheet. The data were collected between 10th July 2024 to 30th November 2024. Gilbert and Lowenstein’s strategies were applied to enhance the accuracy of data collection during medical chart review [26]. The first author reviewed and verified the data collected by the research assistant for every 10th patient, and the data were compared. The research assistant was blinded to the study's hypothesis, aim, and objectives.

### Data analysis

Univariate descriptive analysis was used to summarise and report on the patient and temporal data. We calculated frequencies and percentages for nominal data and examined the median, mode, standard deviation, and quartiles for numerical data. Data were captured and analysed using Microsoft Excel (Version 16.22, Microsoft, Washington) and analysed with IBM SPSS (Version 30.0, SPSS, Chicago). Pearson’s chi-square and Fisher's exact tests were used to compare categorical responses, and the Kruskal-Wallis test was used to compare continous (median time) variables. Statistical significance testing was set at the 95% confidence level, and therefore, a p-value<0.05 denotes statistical significance.

### Ethical considerations

Ethical approval was obtained from the University of Cape Town (UCT) Health Research Ethics Committee (HREC)—HREC 072/2024. A waiver of consent was approved as the study posed minimal risk to patients, using only retrospectively and routinely collected non-identifiable data. Further approvals were obtained from the Western Cape Department of Health and Mitchells Plain Hospital Research Committee (WC_202403_025). All data were deidentified so that patients and staff members managing patients were not identifiable.

## Results

**Fig. 1:**
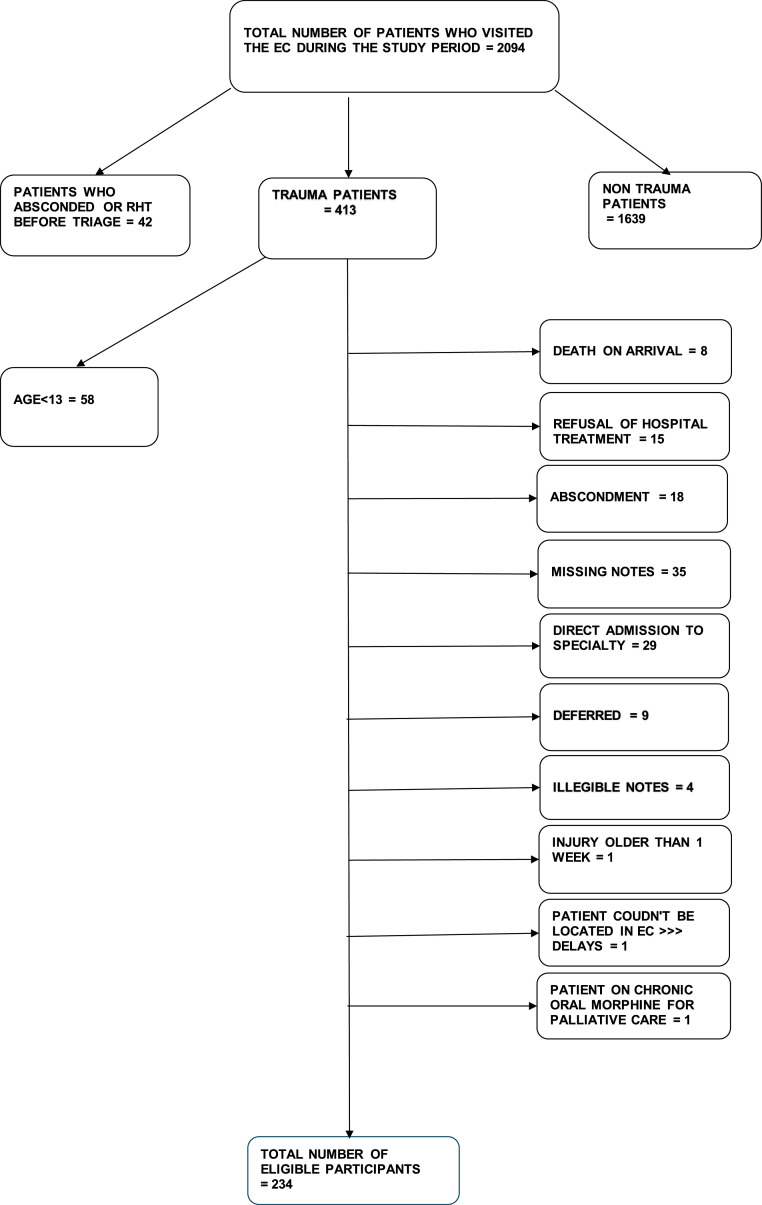
Flow chart of recruitment and exclusion criteria. *RHT refusal of hospital treatment.*

During the study period, 2094 patients visited the EC, with 234 eligible for the study (Fig. 1). Most of these patients were male (73.1%, 171), with a median age of 33 (IQR 26–41) and an age range of 13 to 98. Interpersonal violence accounted for 56.5% of the visits, including stabs, blunt assaults, and gunshot wounds. Most patients were discharged from the EC (76.9%, 180) (Table 1).

**Table 1:** Demographics and clinical information of included trauma patients.

		Total (*n* = 234) count	Analgesia administered (*n* = 99) N (row %)	No analgesia administered (*n* = 135) N (row %)	p- value
Sex	Female	63	30 (47,6)	33 (52,3)	
	Male	171	69 (40,3)	102 (59,6)	0,318
Age	13 – 19	29	6 (20,6)	23 (79,3)	
	20 – 39	134	64 (47,7)	70 (52,2)	
	40 – 59	56	25 (44,6)	31 (55,3)	
	60+	15	4 (26,6)	11 (73,3)	0,32
Mode of Referral	CHC referral	7	5 (71,4)	2 (28,5)	
	GP Referral	7	3 (42,8)	4 (57,1)	
	SAPS	3	2 (66,6)	1 (33,3)	
	self-referral	217	89 (41,0)	128 (58,9)	0,346
Mode of transport	Metro EMS	38	24 (63,1)	14 (36,8)	
	Private	193	73 (37,8)	120 (62,1)	
	SAPS	3	2 (66,6)	1 (33,3)	**0,011**
SATS Triage	Green	22	5 (22,7)	17 (77,2)	
	Yellow	131	44 (33,5)	87 (66,4)	
	Orange	67	41 (61,1)	26 (38,8)	
	Red	14	9 (64,2)	5 (35,7)	**0,001**
Pain score documented (triage)	None	157	70 (44,5)	87 (55,4)	
	Moderate	58	20 (34,4)	38 (65,5)	
	Severe	19	9 (47,3)	10 (52,6)	0,37
Mechanism	Blunt Assault	57	18 (31,5)	39 (68,4)	
	Stab	57	23 (40,3)	34 (59,6)	
	Fall	43	15 (34,8)	28 (65,1)	
	GSW	18	15 (83,3)	3 (16,6)	
	PVA	22	10 (45,4)	12 (54,5)	
	MVA	14	9 (64,2)	5 (35,7)	
	Sport-related injury	9	1 (11,1)	8 (88,8)	
	Other*	12	8(66,7)	4(33,3)	**0,001**
Simplified diagnosis	Open wound	73	29 (39,7)	44 (60,2)	
	Superficial injury	52	16 (30,7)	36 (69,2)	
	Fracture/Dislocation	41	29 (70,7)	12 (29,2)	
	Polytrauma	24	13 (54,1)	11 (45,8)	
	Sprain/strain	23	3 (13,0)	20 (86,9)	
	Head/torso	16	5 (31,2)	11 (68,7)	
	Burns	5	4 (80)	1 (20)	**0,001**
Disposition	Discharged	180	59 (32,7)	121 (67,2)	
	Admitted	41	31 (75,6)	10 (24,3)	
	Transferred	11	7 (63,6)	4 (36,3)	
	Died	2	2 (100)	0 (0)	**0,001**

*Mechanism-other (burns, glass cuts, unspecified); CHC Community Health Centre; GP General Practitioner; SAPS South African Police Service; EMS Emergency Medical Services; GSW Gunshot Wounds; PVA Pedestrian Vehicle Accident; MVA Motor Vehicle Accident; n number of patients; Statistically significant findings are highlighted.

Only 32.9% (77) of the patients had their pain assessed and documented during the triage process. Most patients (97.4%, 75) underwent a VRS pain assessment (no pain/mild/ moderate/ severe pain) as part of the triage process by a triage nurse, recorded on their triage forms as part of SATS. Only two patients had a pain assessment documented by doctors with a numerical rating scale (NRS) pain score (on a scale of 0–10); neither received analgesia. None of the patients had repeat pain assessments. There was no statistically significant difference in receiving analgesia between those with a documented pain assessment and those without (*p* = 0.314). Only 42.3% (99) of patients received analgesia, and we found that patients transported by EMS were more likely to receive analgesia than those who arrived by private transport (*p* = 0.011).

Higher acuity triage patients (red and orange) were more likely to receive analgesia (*p* < 0.001). Only 33.3% (60) of patients who were discharged received analgesia in the EC, compared to 75.6% [31] of admitted patients, which was statistically significant (*p* < 0.001). There were significant statistical differences (*p* = 0.001) in receiving analgesia among patients with different mechanisms of injury and dispositions.

Table 2 shows the pharmacological agents used in patients who received analgesia; no significant association was found between the administered medications and any of the analysed variables. Notably, there was no correlation between pain scores at triage and the type of analgesic received, with a seemingly higher prevalence of patients with no pain assessment and moderate scores among those receiving opioids or ketamine, which are generally reserved for severe pain as per the WHO pain ladder. In contrast, patients with severe pain received NSAIDS or paracetamol.

**Table 2:** Comparisons of analgesic drugs used in various categories of patients.

Drug administration		Ketamine (*n* = 17)	Opiate ^#^ (*n* = 48)	NSAIDS (*n* = 44)	Paracetamol (*n* = 68)
Age	Median (IQR)	30 (23–36)	34 (28–40)	33 (26–41)	34 (28–43)
	Range	13–56	13–58	13–77	13–98
	13 – 19	3	1	4	3
	20 – 39	11	34	27	41
	40 – 59	3	13	11	20
	60+	0	0	2	4
SATS Triage	Green	1	2	3	5
	Yellow	3	20	26	34
	Orange	8	21	15	24
	Red	5	5	0	5
SATS GY vs OR	Green/ Yellow	4	22	29	39
	Orange/ Red	13	26	15	29
Grouped diagnoses	Burns	2	3	1	1
	Fracture/ dislocation	6	12	12	19
	Open wound	5	20	9	19
	Polytrauma	3	6	7	10
	Sprain/strain	0	1	2	1
	Superficial injury	0	3	12	16
	Head/torso unspecified	1	3	1	2
	Blunt Assault	2	6	11	15
Mechanism of injury	Burns	2	3	1	1
	Unspecified	1	0	2	2
	Fall	2	7	4	10
	GSW	1	9	8	14
	MVA	0	5	5	6
	PVA	4	2	5	5
Mechanism of injury	Stab	4	16	7	14
	Others*	1	0	1	1
Simplified Disposition	Admit	10	20	8	17
	Discharged	4	21	36	49
	Died	1	0	0	1
	Transferred	2	7	0	1
Pain score at triage	No pain assessment	14	37	24	45
	Moderate	1	7	14	16
	Severe	2	4	6	7

Others: sport-related injuries, bite wounds, and cuts from glass. SATS: South African Triage Score; NSAIDS: Non-steroidal anti-inflammatory drugs; GSW: Gunshot wound; MVA: Motor vehicle accident; PVA: Pedestrian vehicle accident; GY: Green & Yellow or Orange & Red. Two patients received both IV fentanyl and IV morphine, which are classified as opiates, resulting in a total opiate usage count of 50. The total number of drugs administered is 179. Only 99 patients received analgesia; some patients received multiple analgesic drugs. There was no significant statistical difference in drug administration among all groups.

It is also noteworthy that opioids were used 50 times in 48 patients, with fentanyl combined with other opioids in two cases. Morphine was administered a total of 31 times, with 81% (25) of the doses given intramuscularly (IM) compared to intravenous (IV) administration. (Table 3) The time from arrival to first analgesic administration in EC was documented in 83 patients, with a median of 375 min (IQR 152–611), ranging from 0 min to 1205 min (Table 4) Of note, there was no statistical difference between pain scores and the median time from arrival to analgesia (p=0.102).

**Table 3:** Drugs administered compared to routes of administration.

Drug administration counts (179)		TOTAL	ORAL	IMI	IVI
KETAMINE	IVI ketamine (17)	17	0	0	17
OPIOIDS	IVI morphine (6)	50	9	25	16
	IMI morphine (25)				
	IVI fentanyl (10)				
	Oral tramadol (9)				
NSAIDS	IMI diclofenac (18)	44	26	18	0
	Oral ibuprofen (26)				
PARACETAMOL	IVI paracetamol (8)	68	60	0	8
	Oral paracetamol (60)				

IMI: Intramuscular injection; IVI: Intravenous injection; NSAIDS: Non-Steroidal Anti- Inflammatory Drugs.

**Table 4:** Time from arrival to administration of analgesia.

Delays in Administration of Analgesia		Arrival to Triage (min) (*n* = 234)	Arrival to analgesia (min) (*n* = 83)
Documented time intervals	Median (IQR)	34 (11–72)	375 (152–611)
	Range	0–563	0–1205
Pain score documented Median (IQR)	No score documented	28 (6–69)	334.5 (78–553)
	Moderate	48 (16–79)	514 (300–789)
	Severe	35 (12–68)	401 (333–606)

min: minutes; IQR: interquartile range.

## Discussion

Less than half of trauma patients at a relatively well-resourced and staffed EC in Cape Town received any analgesia in the EC, despite many markers of likely painful presentations, many severe injuries, and a high proportion of patients admitted. The admission and transfer rate (22%) was significantly higher than in another district hospital in Cape Town, where only 6% of trauma patients were admitted or transferred to a higher level of care, indicating that most patients had sustained significant injuries [27].

The study reflected a population cohort with demographics comparable to larger trauma studies in Cape Town, characterised by a predominance of young males, a yellow triage, and interpersonal violence victims, attributed to low socioeconomic environment in the population attending MPH EC [27,28]. Most patients arrived via their own transport and received no prehospital treatment, making the triage area their first point of medical contact. This is crucial, as pain assessment should occur at the first point of contact with patients in the EC to facilitate timely, effective pain management interventions [29].

Pain assessment was documented as part of the SATS triage in less than one-third of patients; almost none had a pain assessment documented by a doctor, and no patients had their pain reassessed. This is concerning, as we know that adequate pain management requires early pain assessment and reassessment to tailor individual pain management using standardised assessment tools such as numeric rating scales (NRS) and visual analogue scales (VAS), both of which are included in MPH EC doctor’s clerking sheet. In this study, HCPs predominantly used a verbal rating scale driven by the triage process, where moderate or severe pain acts as a discriminator, placing them in a higher triage category (76). These findings support other studies conducted locally in various settings where pain assessment was poor [7,18,30].

A study conducted in KwaZulu-Natal also indicated a lack of formal pain assessment among patients admitted with long bone fractures from the EC, resulting in suboptimal pain management [9]. This problem is not exclusive to South Africa. A study in Guinea found that only three out of 880 patients had their pain assessed [8]. Internationally, barriers to pain assessment and management include insufficient knowledge, inadequate training, overcrowding, and lack of written protocols for pain assessment and management in many ECs [31,32].

We found that only 42.3% (99) of patients received analgesia during their stay in the EC, with a median waiting time of 375 min. These figures are concerning, given that access to pain management is a human right, as declared by the WHO and Human Rights Watch [33]. A prehospital study in Cape Town demonstrated that pain management was poor, with only 18% of trauma patients receiving analgesia. Studies from Tanzania and Rwanda show that only 46.3% and 54.8% of patients received analgesia [34,35]. This is a global issue, even in well-resourced settings such as Canada, where a 2018 study revealed that although 74.2% of patients had their pain assessed at triage, only 24.9% of those patients received analgesia, with a median waiting time for analgesia of 95 min [36]. Although this study was not powered to assess the adequacy or appropriateness of analgesia, we note with concern the lack of congruence between pain assessments (when performed) and the type of analgesia given. Ketamine and opiates, which would be regarded as “stronger” analgesics according to the WHO ladder, appeared to have been used more with those having no pain assessment or moderate scores [17].

Of particular concern are vulnerable populations, such as older adults and teenagers, few of whom received analgesia in the EC (26.7% and 20.7%, respectively). None of the older adults (60+ years) received opiates, and few received NSAIDs. This may be attributed to the fear of adverse effects related to opioids in this group because of comorbidities, polypharmacy, and ageing physiology. A multimodal analgesic approach that incorporates both pharmacological and non-pharmacological strategies is recommended. With judicious use, opioids remain the standard of care for severe pain even in older patients [37].

Among the patients who received opioids, the IM administration of morphine was the predominant route in this study. This is despite IV opioid administration being the gold standard in acute severe pain, especially in trauma, where peripheral tissue absorption may be impaired, and there is a lack of ability to titrate the medication to effect and unpredictability associated with IM route [38]. A French study suggested IV morphine was underprescribed due to overcrowding, the need for venous access and IV titrations being a human resource-intensive factor, the reluctance of junior doctors to prescribe morphine and the lack of space for ambulatory patients to be safely monitored [33].

We note that methoxyflurane was unavailable at this study site, and there was no evidence of regional nerve blocks being utilised, despite both being shown to be safe and effective [37]. Although delay metrics paint a picture of an overcrowded EC where patients of all acuities experience delays in being seen, there is no reason for patients already assessed at triage to wait in pain. Our study highlights the delays in receiving analgesia, even for patients with severe pain. Many international guidelines recommend that patients with severe pain receive appropriate analgesia within 15–30 min of arrival at the EC [[39], [40], [41]]. Our study patients with severe pain had to wait for a median time of 400 min, so there is a clear gap in the recommended versus actual practices, which has profound implications [25]. This can be attributed to many factors, including systemic factors such as overcrowding, knowledge deficits and poor attitudes towards pain management by HCPs [17,18,42].

Despite research showing the impact of poor pain management, there remains a significant inadequacy in the effective and early management of pain. Systemic inefficiencies include overcrowding in ECs, a shortage of staff relative to the patient volume, a lack of qualified personnel to administer certain medications such as IV drugs, restrictions on opioid use due to fear of addiction and abuse, and the unavailability of drugs [17,18,31,32]. These issues are particularly pronounced in LMICs.

Factors influencing HCPs include inadequate knowledge, negative attitudes, and insufficient training or education regarding pain, underscoring the urgent need for structured pain management protocols [31,32]. Patient factors encompass cultural perspectives, as some individuals may be reluctant to express their pain and face language barriers that can lead to miscommunication with healthcare providers. Additionally, some patients might decline pain medication because of misinformation, particularly concerning opioids, highlighting the necessity for patient education on pain management [31,32]. Pain is often deprioritised in emergencies, where life-threatening conditions take precedence. Some patients present in haemodynamically unstable conditions and are unable to communicate their pain effectively.

The lack of evidence-based standardised pain management protocols is a significant barrier to effective pain management. Implementing these protocols is crucial in addressing the ongoing challenge of inadequate pain management in LMICs, particularly in high-pressure environments like ECs [32,43]. Nurse-led analgesia protocols have been shown to be safe, enhance the quality of patient care, and facilitate prompt pain treatment [44]. The implementation of these written protocols will require teamwork, better access to analgesic drugs at triage, and an improved understanding of pain assessment and management among nurses, empowering them to lead pain management, as they are typically the first point of contact in the triage area [32]. Inter-professional collaboration can enhance protocol implementation in the EC, particularly locally, where inadequate pain assessment and management, compounded by long waiting times for analgesia administration, are evident [43].

## Limitations

Challenges included missing or poorly documented data, typical of chart reviews. Non-pharmacological strategies were not investigated, and poor documentation by healthcare providers —a known issue —could not be addressed. While the study offers insight into local practices from a single site, it may also serve as a foundation for improving pain management and initiatives, such as triage nurse-led analgesia protocols.

## Conclusion

The prevalence of acute trauma pain is high in this EC setting, where early assessment and timely pain management are crucial. We found that trauma patients experienced inadequately managed pain, which has profound implications for the quality of care and patient satisfaction, as well as recovery. This issue is complex due to systemic inefficiencies, HCPs and patient factors, all compounded by a lack of structured pain management protocols. This underscores the need for enhanced training for HCPs, especially triage nurses, who are the first point of contact with patients in pain. Nurse-led analgesia protocols have been shown to be effective, safe, and to improve the overall management of pain in ECs. Future research should examine the factors contributing to poor pain assessment and management in this setting, delays in administering analgesia, and the feasibility of implementing early, nurse-led analgesia.

## Dissemination of results

The results of this study will be shared with stakeholders at Mitchell’s Plain Hospital and the University of Cape Town Emergency Medicine Division through presentations at conferences and in peer-reviewed publications.

## Funding

The primary author (NN) funded this study, in addition to funding for a research assistant from the Division of Emergency Medicine, University of Cape Town.

## CRediT authorship contribution statement

**Ngcebo Ndebele:** Conceptualization, Methodology, Formal analysis, Project administration, Writing – original draft. **Laverne Phillips:** Data curation, Writing – review & editing, Supervision. **Peter Hodkinson:** Conceptualization, Methodology, Formal analysis, Writing – review & editing, Visualization, Supervision.

## Declaration of competing interest

The authors declare that they have no affiliations or involvement with any organisation or entity with financial interests in the subject matter or materials discussed in this manuscript. PH is an editor of the journal, but was not involved in editorial process or decisions for this submission.
